# Nutraceuticals and Pain Disorders of the Gut–Brain Interaction in Infants and Children: A Narrative Review and Practical Insights

**DOI:** 10.3390/nu16030349

**Published:** 2024-01-25

**Authors:** Silvia Salvatore, Mariagrazia Carlino, Simona Sestito, Daniela Concolino, Massimo Agosti, Licia Pensabene

**Affiliations:** 1 Pediatric Department, Hospital “F. Del Ponte”, University of Insubria, 21100 Varese, Italy; silvia.salvatore@uninsubria.it (S.S.); massimo.agosti@uninsubria.it (M.A.); 2 Pediatric Unit, Department of Medical and Surgical Sciences, Magna Graecia University of Catanzaro, 88100 Catanzaro, Italy; mariagraziacarlino08@gmail.com (M.C.); sestitosimona@unicz.it (S.S.); dconcolino@unicz.it (D.C.)

**Keywords:** nutraceutical, complementary therapies, herbs, fibers, prebiotics, probiotics, abdominal pain, irritable bowel syndrome, infantile colic, FGIDs, FAPs, disorders of the gut–brain interaction

## Abstract

Different nutraceuticals are often considered by parents of infants and children with abdominal pain and disorders of the gut–brain interaction. Herb extracts and natural compounds have long been used in traditional medicine, but clinical pediatric trials are very limited. This narrative review based on relevant studies identified through a search of the literature in Pubmed and Medline updated to October 2023 focused on the effect of nutraceuticals in infantile colic, functional abdominal pain, and irritable bowel syndrome in children and adolescents. Significant reductions in colic episodes and crying time were reported in two studies on fennel (seeds oil or tea), in three studies on different multiple herbal extracts (all including fennel), in one study on *Mentha piperita*, and in at least two double-blind randomized controlled studies on *Lactobacillus reuteri* DSM 17938 and *Bifidobacterium lactis* BB-12 (10^8^ CFU/day for at least 21 days) in breast-fed infants. Compared to a placebo, in children with functional abdominal pain or irritable bowel syndrome, a significant reduction in pain was reported in two studies supplementing peppermint oil capsules or psyllium fibers, and in one study on corn fiber cookies, partial hydrolyzed guar gum, a specific multiple herbal extract (STW-5), or vitamin D supplementation. To date, there is moderate-certainty evidence with a weak grade of recommendation on *Lactobacillus reuteri* DSM 17938 (10^8^ CFU/day) in reducing pain intensity in children with functional abdominal pain and for *Lactobacillus rhamnosus* GG (1–3 × 10^9^ CFU twice daily) in reducing pain frequency and intensity in children with IBS. Further large and well-designed pediatric studies are needed to prove the efficacy and safety of different herbal extracts and prolonged use of studied products in infants and children with pain disorders of the gut–brain interaction.

## 1. Introduction

“Nutraceutical” is a term introduced by Stephen Defelice in 1989 and identifies “a food or part of a food or a dietary supplement, that has a medical or health benefit, including the prevention and treatment of disease” [1].

The medicinal and spiritual applications of plants, herbs, and other natural compounds date back to many centuries earlier and were already reported in Egyptian, Roman, Mesopotamian, and Greek civilizations and depicted in some ancient artworks [2].

In ancient Indian literature, Susruta and Charaka mention the use of cardamom, turmeric, ginger, cinnamon, and pepper for their medicinal properties. Some of these spices continue to be used for the treatment of multiple conditions, including abdominal pain disorders, throughout the world [3]. The concept of nutraceuticals has evolved over time but still does not have a well-established definition worldwide [4]. Nutraceuticals have been recently classified into traditional and non-traditional nutraceuticals. Traditional nutraceuticals include three subcategories: chemical constituents (nutrients, herbals, phytochemicals, polyunsaturated fatty acids); probiotics and prebiotics; and nutraceutical enzymes. The non-traditional nutraceuticals are fortified nutraceuticals and recombinant nutraceuticals [5]. Nutraceutical compounds are generally considered as products, including minerals, vitamins, amino acids, vegetables, or herbs, that confer benefit to human health by improving functional, mental, and physical activities [6].

Several in vitro and in vivo studies have demonstrated nutraceuticals’ efficacy on numerous patho-mechanisms targeting gastrointestinal smooth muscles, visceral afferent nerves, inflammation, gut permeability, and the gut microbiome [7,8,9,10,11].

Despite their wide and long-term use, scientific information about nutraceuticals is limited: there are very few studies evaluating their safety [12] and efficacy [13,14,15,16], and there is no international regulation regarding their marketing and dosing [5,17].

Functional abdominal pain (FAP) disorders are disorders of the gut–brain interaction (DGBI), and, according to Rome IV criteria, include infantile colic, irritable bowel syndrome, functional dyspepsia, and functional abdominal pain not otherwise specified [13,18,19].

They affect more than 20% of infants and children [20] throughout the world, with a significant economic impact, decreased quality of life, and long-term adverse outcomes [21].

According to data published in 2015 from a multicenter trial in The Netherlands, the total annual costs per patient with FAPs, calculated as the sum of medical and non-medical costs, were estimated to be more than EUR 2500, excluding initial diagnostic investigations [22].

Despite an increasing recognition of multiple underlying factors, treatment is still challenging and symptoms often persist despite multiple non-pharmacological and pharmacological interventions, including probiotics, diets, spasmolytics, anti-depressants, and cognitive–behavioral approaches [23]. Because of the frequent lack of benefit of conventional treatments, almost 40% of parents of pediatric gastroenterology patients search for complementary and alternative medicine (CAM) for their child. More than 90% of these parents also considered it important for pediatricians to acquire knowledge of and initiate CAM research [24].

Likewise, a large number of adult patients with functional gastrointestinal disorders use CAMs [25].

The scope of this narrative review is to summarize current evidence of the effects of nutraceuticals on abdominal pain disorders in infants and children and to provide health care professionals a practical guide for their possible clinical application.

## 2. Literature Search Strategy

Medline-PubMed was searched up to 31 October 2023 using the following MESH and Boolean terms: “herbs” OR “herbal supplements” OR “nutraceuticals” OR “ginger” OR “iberogast” OR “STW-5” OR “peppermint” OR “*Mentha piperita*” OR “licorice” OR “liquorice” OR “fennel” OR “vitamins” OR “antioxidants” OR “polyphenols” AND (“functional gastrointestinal disorders” OR “abdominal pain” OR “infantile colic”), limited to English language and children (0–18 years). References of selected studies and reviews as well as a Google database search were also used to retrieve additional original studies.

## 3. Nutraceuticals and FAP/DGBI in Pediatric Age

Only a very limited number of nutraceuticals have been assessed in clinical trials in infants and children with FAP/DGBI disorders.

Recent reviews have extensively covered studies evaluating fibers [26,27], probiotics, prebiotics, and synbiotics in different pediatric gastrointestinal disorders [13,28,29].

Anheyer et al. [30] in 2017 reviewed fourteen trials with 1927 children suffering from acute and functional gastrointestinal disorder: even if evidence was found for peppermint oil in decreasing the duration, frequency, and severity of pain in children suffering from undifferentiated FAP and evidence for effectiveness was found for different fennel preparations (e.g., oil, tea, herbal compound) in treating children with infantile colic, the authors confirmed the need for further clinical studies to be carried out strictly to demonstrate the efficacy and safety of nutraceuticals.

This review focuses on nutraceuticals other than biotics and fibers in infants and children with infantile colic, functional dyspepsia, functional abdominal pain, and irritable bowel syndrome. A summary of studies evaluating these included nutraceuticals in the above disorders in pediatric patients are reported in Table 1.

**Table 1:** Summary of pediatric studies evaluating nutraceuticals as herbal extracts in infants and children with FAP/DGBI.

Disorder	Nutraceutical	Intervention	Population	Assessment	Outcome	Reference
*Infantile colic*	Fennel seed oil emulsion	Fennel oil vs. placebo	125 infants (2–12 weeks)	Wessel’s criteria and weekly crying time diary	Colic resolved in 65% vs. 23%	[31]
	*Fennel tea*	Herbal tea (fennel tea) 35 mL × 3/day vs. control group	35 infants (age 4–12 weeks)	Wessel’s criteria and daily crying diary	↓ Daily crying time (−1.51 h/day vs. −0.09 h/day in control group)	[32]
	*Matricaria chamomilla*, *Verbena officinalis*, *Glycyrrhiza glabra*, *Foenieulum vulgare*, and *Melissa officinalis* plus glucose	Herbal tea vs. placebo (max 150 mL tds during colic, for 7 days)	68 infants (2–8 weeks)	Wessel’s criteria and crying diary	↓ colic in 57% vs. 26%	[33]
	*Matricariae recutita*, *Foeniculum vulgare*, and *Melissa officinalis*	Herbs agent vs. placebo (2 mL/kg/day) × 7 days	88 breastfed infants	Wessel’s criteria and daily crying time diary	↓ crying time (−124.3 min vs. −28.8 min); ↓ colicky infants (85% vs. 49%,)	[34]
	*Carbo vegetabilis*, *Prunus spinosa*, *Carum carvi*, *Matricaria chamomilla*, *Foeniculum vulgare*, *Zingiber officinale*, *Melissa officinalis*, and *Mentha piperita*	1.25 mL during a colic episode and repeated after 120 min, if needed	30 infants (age 3–16 weeks)	Wessel’s criteria and modified Barr’s crying diary	↓ ≥50% in 73% of infants by day 7 and in 80% by day 14	[35]
	Leaves of *Mentha piperita* or simethicone	*Mentha piperita* (1 drop/kg) vs. simethicone (2.5 mg/kg) daily for 7 days	30 infants (15–60 days)	Wessel’s criteria, daily crying diary and measured with a chronometer	↓ in 40% infants on *Mentha piperita* and in 43% on simethicone	[36]
*IBS*	Peppermint oil capsules	1–2 cps (187–374 mg) × 3/day × 2 weeks vs. placebo (arachis oil)	42 children (8–12 years)	Symptom scales	↓ symptoms (71% vs. 43%); ↓ severity (79% vs. 16%); no changes in GSRS	[37]
*FAP*	Peppermint oil (Colpermin)	1–2 cps (187–374 mg) × 3/day × 1 month vs. symbiotic Lactol (*Bacillus coagulans* + FOS) vs. placebo	120 children (4–13 years)	Symptom scales	Significant ↓ in pain duration; ↓ severity vs. placebo and lactol; ↓ frequency vs. placebo	[38]
*FGIDs (43% IBS; 26% FD)*	STW-5	10–20 drops, 3/day for 1 week	980 children (3–14 years)	Adapted GIS score	↓ GIS of 76%	[39]
*Functional Dyspepsia*	STW-5 (Iberogast)	Dose not reported (10% added diet + other treatment)	50 out of 154 children (4–18 years)	Symptom recording	↓ duration of symptoms in males	[40]

Legend: FAP = functional abdominal pain; FD = functional dyspepsia; FGIDs = functional gastrointestinal disorders; FOS = fructooligosaccharides; GIS = gastrointestinal symptom score; GSRS = Gastrointestinal Symptom Rating Scale. IBS = irritable bowel syndrome; ↓ = reduction.

## 4. Fennel

Fennel, taxonomically identified as Foeniculum vulgare, is a perennial plant used, in various forms, in traditional medicine for ages. The main component of fennel is anethole, also known as estragole, which is extracted from its seeds and has a chemical structure similar to dopamine, with a reported relaxing effect on the intestinal smooth muscles [41].

Two studies reported a beneficial effect of fennel on crying in colicky infants.

In 2003, a randomized placebo-controlled trial tested the effect of fennel seed oil emulsion in 125 infants (2 to 12 weeks of age) with infantile colic [31].

Colic, as defined according to Wessel’s criteria, disappeared in 65% (40/62) of infants in the fennel group compared to 23% (14/59) of the placebo group (*p* < 0.01). A significant reduction in weekly crying time was also found in the treatment group compared with the placebo group (Absolute Risk Reduction (ARR) = 41% (95% CI 25 to 57), Number Needed to Treat (NNT) = 2 (95% CI 2 to 4)). No side effects were reported in the two groups of infants [31].

A prospective randomized controlled study involved 35 colicky infants who were treated with herbal (fennel) tea (35 mL three times a day for 7 days). Crying time significantly decreased from 5.11 ± 1.43 h/day at baseline to 3.20 ± 1.23 h/day after one week of intervention (−1.51 h/day vs. −0.09 h/day in the control group, *p* < 0.001) [32].

## 5. Ginger

Ginger is the rhizome of the *Zingiber officinale* commonly used as food and in Asian traditional medicine for gastrointestinal symptoms [42].

Ginger contains many different compounds, such as carbohydrates, lipids, terpenes, phenolic components, 6-gingerol,8-gingerol,10-gingerol, and 6-shogaol, which may act through cholinergic and calcium antagonist mechanisms, M3 and 5-HT3 receptors, and the synthesis of prostaglandins [43,44].

Ginger has been studied mostly for nausea, in pregnancy [45], in adults and children on chemotherapy [46,47], or post surgery [48].

Aregawi et al. [49] showed, in a before-and-after clinical study on an adult population affected by FD, a considerable improvement in their condition after four weeks of ginger supplementation, with highly statistically significant findings in postprandial fullness (*p* = 0.033, 95% CI = 0.01–0.26), early satiety (*p* = 0.001, 95% CI = 0.10–0.37), epigastric pain (*p* = 0.000, 95% CI = 0.16–0.42), epigastric burning (*p* = 0.003, 95% CI = 0.10–0.45, and heartburn (*p* = 0.209, 95% CI = −0.04–0.20).

Wu et al. showed that ginger accelerates gastric emptying and stimulates antral contractions in healthy adult individuals [50].

One study reported an antiemetic effect in children with gastroenteritis [51], but we did not find studies evaluating ginger in FAPs or DGBI in children.

Nonetheless, a survey about the use of complementary and alternative therapies in 100 children with abdominal pain disorders found that 11% of children with IBS used ginger as treatment [52].

Ginger is generally considered safe. The most common adverse effects, at the dosage above 5 g per day, are mouth and throat irritation, abdominal discomfort, heartburn, burping, and diarrhea. These symptoms may be avoided or reduced by taking ginger in capsule form [53].

## 6. Licorice

Licorice root (*Glycyrrhiza glabra*) is an ancient herb mostly used in Chinese medicine. It contains triterpentoid saponin glycyrrhizin, flavonoids, isoflavonoids, chalcones, cumarins, triterpenoids, and sterols, which have anti-inflammatory, immune, metabolic, endocrine, and possible gastric activities [54].

After more than 2 weeks of intake, non-deglycyrrhizinated licorice can cause hypertension and hypokalemia via a mineralcorticoid effect (sodium and water retention) [55].

There are currently no pediatric studies evaluating licorice as single supplementation in abdominal pain disorders.

## 7. Peppermint

Peppermint is a species of mint, a perennial herb of the Lamiaceae family, used for ages for its presumed anti-inflammatory, analgesic, and antispasmodic effects [37,56,57].

Peppermint oil, obtained via steam distillation from the fresh leaves, is reported to induce smooth muscle relaxation (by blocking calcium channel [58] or direct enteric nervous system effects); modulate visceral sensitivity (via transient receptor potential cation channels) and psychosocial distress; and exert anti-microbial and anti-inflammatory effects [59].

Among the main compounds of peppermint are flavonoids and phenolic acids.

Moreover, menthol may act on Cajal interstitial cells, stimulate the production of prostaglandins, and have a nociceptive action in the brain through the activation of GABA receptors type A [60].

A double-blind randomized crossover study enrolling 30 colicky infants showed that episodes of colic decreased in infants treated with *Mentha piperita* and with simethicone (from 3.9 per day at baseline to 1.6 per day after one week of intervention), the duration of crying was similar between the two groups of treatment, and colic disappeared in 40% of infants treated with *Mentha piperita* vs. 43% of infants on simethicone [36].

In one pediatric randomized, double-blind, placebo-controlled study [37], peppermint oil enteric-coated capsules (1–2 capsules containing 187 mg, three times per day) were administered to 42 children (aged 8–12 years) with IBS for two weeks. The intervention group reported more frequently reduced symptoms (71% vs. 43%) and severity (79% vs. 16%) but no significant changes in Gastrointestinal Symptom Rating Scale.

Another randomized placebo-controlled trial compared the effects of peppermint oil (1–2 capsules of 187 mg, three times per day) (34 children), a symbiotic (*Bacillus coagulans* and fructooligosaccharide) (29 patients), and a placebo (folic acid) (25 patients) for one month in children with FAPs (30 with IBS). The group who received peppermint oil had a significant reduction in the severity of pain (as assessed on a one to ten scale), and the duration (minutes per day) and frequency of abdominal pain (episodes per week) compared to the placebo and compared to the symbiotic [38].

No adverse events were noted. Two other studies performed in children with abdominal pain disorders pointed out that peppermint oil has no effect on microbiome composition [61] or small bowel/colonic transit time [62].

## 8. Multiple Herbal Extracts

A prospective double-blind randomized controlled study including 68 colicky infants showed that colic disappeared in 19/33 (57%) infants taking herbal tea (up to 150 mL three times per day during episodes of colic, for 7 days) vs. 9/35 (26%) infants in the placebo group (*p* < 0.01). The tea contained extracts of chamomile (*Matricaria chamomilla*), vervain (*Verbena officinalis*), licorice (*Glycyrrhiza glabra*), fennel (*Foenieulum vulgare*), and balm mint (*Melissa officinalis*) plus glucose and the placebo was made with glucose and natural flavors [33].

A randomized, double-blind, placebo-controlled trial investigated the effect of a phytotherapeutic agent (combination of *Matricariae recutita*, *Foeniculum vulgare*, and *Melissa officinalis*) in 88 colicky breastfed infants. After one week of intervention, crying time was significantly reduced in more infants treated with the combination of herbs than in infants in the placebo group (85% vs. 49%, *p* < 0.005). The daily average crying time also decreased significantly more in the intervention group than in the placebo group (−124.3 min vs. −28.8 min, *p* < 0.005), and these results were maintained fifteen days after the end of therapy (average crying time 82.1 min/day vs. 165.3 min/day, *p* < 0.005), No side effects were reported [34].

A recent open-label single-group study was conducted in 30 colicky infants (age 3–16 weeks) evaluating the effect of a product containing *Carbo vegetabilis* (vegetable charcoal), *Prunus spinosa* (Blackthorne), *Carum carvi* (Caraway), *Matricaria chamomilla* (Chamomile), *Foeniculum vulgare* (Fennel), *Zingiber officinale* (Ginger), *Melissa officinalis* (Lemon Balm), and *Mentha piperita* (Peppermint). The product was administered 1.25 mL orally during a colic episode and repeated after 120 min, if needed, Average daily crying time was recorded using a modified Barr’s diary and analyzed after 7 and 14 days from recruitment. Daily crying time and flatulence significantly decreased (*p* < 0.05) with a reduction of ≥50% in 73% of infants by day 7 and in 80% by day 14. After stopping the intervention, 40% of infants had a relapse of colic [35].

## 9. STW-5 (IBEROGAST)

STW-5 is an herbal combination of nine alcoholic extracts from *Iberis amara, Angelicae radix, Cardui mariae fructus, Chelidonii herba, Liquiritiae radix, Matricariae flos, Melissae folium, Carvi fructus,* and *Menthae piperitae* [39].

This combination is supposed to have a synergic action on reducing gastrointestinal contraction and inflammation and stimulating gastric secretion [63].

STW-5 was introduced to the market in the 1960s and in the last 30 years clinical trials have been conducted [64,65].

Among the nine herbal extracts, Iberis amara selectively inhibits muscarinic M3 receptors, while chelandine herb and chamomile flowers act on 5-HT4 and licorice root on 5-HT3 receptors.

In the esophagus, it acts as a mucosal protector by increasing mucin and prostaglandin E2 secretion and increases the pressure of the LES (lower esophageal sphincter) [66].

In the stomach, STW5 relaxes the fundus and corpus and increases antral region contractions [67,68].

A prospective observational study included 980 children (age 3–14 years) with FGID (IBS in 43%, FD in 26%). STW-5 was administered in 10–20 drops three times a day for 1 week. During the treatment period, an adapted gastrointestinal symptom score (GIS) decreased 76% from baseline score and 39% percent of children reported complete relief of their symptoms, with similar effects among different patient groups. Seven patients (0.7%) reported adverse events (skin rash, nausea, vomiting, abdominal pain, and increased gastrointestinal complaints) [39].

In a retrospective study on 154 children (aged 4–18 years) affected by functional dyspepsia, STW5 showed significant benefit only in boys [40].

## 10. Fibers and Prebiotics

In recent decades, many dairy companies have added fibers/prebiotics, such as short-chain galactoligosaccharides and long-chain fructooligosaccharides, to infant formulas to improve infant gut microbiota composition and possibly ameliorate digestive disturbances. However, since these formulas also present hydrolyzed proteins, reduced lactose content, modified fat, and, eventually, probiotics, the clinical benefit in reducing crying and colic, reported in some studies, cannot be clearly correlated to the fibers [26].

We identified only two studies evaluating the effect of fibers in infantile colic [69,70].

Soy polysaccharide was supplemented in a specific infant formula and assessed in 27 colicky infants (aged 2–8 weeks) in a placebo-controlled crossover trial for 9 days. No significant difference in daily crying time and fussing was found between the two groups [69].

In the other randomized double-blind controlled study, 94 infants received galactoligosaccharides and polydextrose or a specific strain of probiotic (Lactobacillus GG 10^9^ colony-forming units/d in 1 dose from day 1 to day 30 and 10^9^ colony-forming units twice daily from day 31 to 60 day) or a placebo for the first two months of life. The group receiving prebiotics and probiotics reported significantly less frequent excessive crying compared to the placebo group (19% and 19% vs. 47%, *p* = 0.02) [70].

We refer to three recent reviews [20,26,27] for a detailed analysis of the role of fibers in children with abdominal pain and gastrointestinal disorders.

In brief, in children with FAPs, corn fiber cookies for two weeks [71] significantly reduced the frequency of abdominal pain compared to placebo. Likewise, partial hydrolyzed guar gum supplementation in 60 children with chronic abdominal pain or IBS significantly decreased IBS score, while improving stool consistency, compared to the placebo group [72].

Conversely, glucomannan did not show any significant benefit on abdominal pain [73].

In another randomized controlled trial on 71 children with IBS, inulin for 4 weeks was less effective than probiotics and synbiotics [74].

Psyllium fiber (6–12 g/day for 6 weeks) [75] reduced the mean number of abdominal pain episodes but not pain intensity.

More recently, the same dosage of psyllium supplementation significantly improved the IBS severity scoring scale and showed a higher remission rate compared to placebo [76].

## 11. Probiotics

As reported by the 2023 ESPGHAN position paper on probiotics for the management of pediatric gastrointestinal disorders [28], in infantile colic there is a moderate certainty of evidence and a weak grade of recommendation for *Lactobacillus reuteri* DSM 17938 and *Bifidobacterium lactis* BB-12 (10^8^ CFU/day for at least 21 days) in breastfed infants. No recommendation can currently be made for or against probiotics in formula-fed infants or for preventing infantile colic. There is also a moderate certainty of evidence and a weak grade of recommendation for *Lactobacillus reuteri* DSM 17938 (10^8^ CFU/day) for reducing pain intensity in children with FAPs and for *L. rhamnosus* GG (1–3 × 10^9^ CFU twice daily) for reducing pain frequency and intensity in children with IBS [28].

## 12. Synbiotics

The recent ESPGHAN position paper on the use of synbiotics for the management of children with gastrointestinal disorders [77] concluded that currently no recommendation can be formulated on the use of any specific symbiotic preparation in the treatment of infant colic, FAP, or IBS.

## 13. Vitamins and Anti-Oxidants

We identified only one pediatric study assessing the effect of vitamin on abdominal pain in children. A randomized controlled trail in 112 adolescents with IBS and vitamin D deficiency showed that oral vitamin D_3_ 2000 IU/day for 6 months normalized the vitamin D level and significantly improved different IBS symptom scores (IBS-SSS, IBS-QoL and total score) compared to the group receiving placebo [78].

Vitamin D may act by reducing inflammation through immune modulation and by regulating the synthesis of serotonin [79].

## 14. Adverse Effects (AEs) of Nutraceuticals in Infants and Children with FAP/DGBI

Nutraceuticals are generally considered safe when used appropriately and in recommended doses. However, it is crucial to recognize that adverse effects can occur, and caution should be exercised, especially in children and adolescents. Dosages recommended for adults may not be suitable for younger individuals. Additionally, the regulation of nutraceuticals can vary, and not all products undergo rigorous testing for efficacy and safety, particularly in the long term; there may be variations in quality among different brands and formulations, and some may contain ingredients that are not appropriate for children. Moreover, high doses of certain nutraceuticals (vitamins and minerals) can lead to toxicity.

The pediatric studies analyzed in this review and reported in Table 1 showed no significant side effects or toxicity with the evaluated nutraceuticals and at the reported doses.

Indeed, no adverse effects were reported throughout a trial of fennel [31] and three of peppermint [36,37,38], as well as in two other studies with multiple herbal extracts [33,34]. Evans et al. [35] conducted an open-label study with 1.25 mL of a multiple herbal extract (*Carbo vegetabilis*, *Prunus spinosa*, *Carum carvi*, *Matricaria chamomilla*, *Foeniculum vulgare*, *Zingiber officinale*, *Melissa officinalis*, and *Mentha piperita*) administered orally (up to 6 doses in 24 h if required) during a colic episode and they described as the only adverse event a febrile episode that resolved spontaneously in 1 child out of 32 enrolled.

Michael et al. [39] reported adverse effects in 0.7% of 1032 children (7 cases) who were treated with STW-5. Six of them discontinued therapy before visit 2 because of these events. These AEs (skin rash, nausea, vomiting, and abdominal pain) were not severe and resolved spontaneously. In one patient presenting tendovaginitis (left arm), any causality was assessed. Only one serious adverse effect was reported (appendicitis), but evaluated as a non-drug-related [39].

In another study of STW-5, no serious adverse events nor clinically relevant changes in laboratory parameters were reported during the study period. There were no significant differences in the proportion of patients reporting non-serious adverse events between both treatment groups [80].

Moreover, the safety profile of STW 5 was extensively evaluated in pre-clinical and in controlled and non-interventional or retrospective clinical studies [64]. The incidence was 0.04% and the adverse drug reactions documented were (in alphabetical order) abdominal cramps, abdominal pain, alopecia, bronchitis, constipation, diarrhea, dizziness, gastrointestinal complaints increased, gastrointestinal pain, hypersensitivity, hypertension, nausea, esophagitis, pruritus, skin rash, sore throat, stomatitis, and vomiting. No serious adverse drug reactions occurred and the studies also found no clinically relevant deviations in laboratory values. Hypersensitivity reactions may occur very rarely and may take the form of pruritus, dyspnea, or skin reactions in pre-disposed patients [81,82].

Doses above 5 g per day of ginger (generally considered safe in the amounts typically found in foods, with few adverse effects reported in clinical trials) have been reported as more likely to cause side effects [53]. The most common adverse effects are irritation of the mouth and throat, abdominal discomfort, heartburn, burping, and diarrhea and sometimes constipation. Ginger bolus from insufficiently chewed pieces of raw ginger, leading to small bowel obstruction, has been reported in four cases [53].

In terms of licorice, after more than 2 weeks of intake, non-deglycyrrhizinated licorice can cause hypertension and hypokalemia via a mineralcorticoid effect (sodium and water retention) [55].

It has been reported that peppermint oil (generally well tolerated at the commonly recommended dosage) can worsen reflux symptoms because of its effects on the lower esophageal sphincter [53] so should be avoided in patients with hiatal hernia or gastroesophageal reflux disease (GERD).

Regarding the adverse effects of fibers [26], only mild side effects such as diarrhea, abdominal distention, flatulence, and vomiting were observed in the experimental group in four studies [83,84,85,86], but no serious adverse events have been reported. Partial or complete fermentation of fibers, resistant to digestion and not absorbed in the human small intestine, occurs in the large intestine, creating flatulence, diarrhea, abdominal distension, and discomfort, depending on the quantity and type of fibers, other food components, the gut microbiota, and individuals’ response and sensitivity [26]. Therefore, excessive fiber intake during childhood should be avoided to limit not only intestinal fermentation but also a possible inadequate energy intake due to increased satiety and reduced bioavailability of minerals. High phytate and oxalate contents of many sources of fibers decrease iron bioavailability [87].

Regarding probiotics, it is well known that their properties are strain-specific, and this applies both for efficacy and safety. Most probiotic strains (including those evaluated in this review) are generally considered safe by the FDA. No adverse effects of the probiotic strains tested in the studies in children with FGIDs were reported. Most AEs and serious AEs were considered unrelated to the study product. However, safety concerns remain in vulnerable subjects, particularly in neonates born prematurely or with short bowel syndrome or immune deficiency [88].

Further studies of major cohorts of patients are needed to establish more accurately the safety profile of the described nutraceuticals, particularly in the long term. Therefore, while some nutraceuticals may have potential benefits for children and adolescents with functional gastrointestinal disorders, it is crucial to approach their use cautiously. Consulting with healthcare professionals who specialize in pediatric gastroenterology is essential for a personalized and safe approach to managing gut–brain interaction disorders in this population.

Caution and medical supervision on the use of nutraceuticals in pediatric subjects have also been advocated in other recent reviews [13,15,20,27].

## 15. Limitations of This Review

We are aware of some limitations of this review. First, we did not perform a systematic review and we limited the literature search to Medline and the English language. However, we retrieved additional studies through references of selected articles and recent reviews as well as a Google database search to reduce the risk of missing original studies. Second, many herbal extracts have long been used in traditional medicine but data are not reported in clinical trials. Third, we aimed to help healthcare professionals orienting in the nutraceutical field with children who are affected by pain disorders of the gut–brain interaction but we did not provide any clear recommendation. Likewise, the European Medicine Agency currently does not recommend any herbal product for gastrointestinal disorders in the pediatric population because of a lack of adequate data on efficacy and safety [89].

## 16. Conclusions

**Figure 1:**
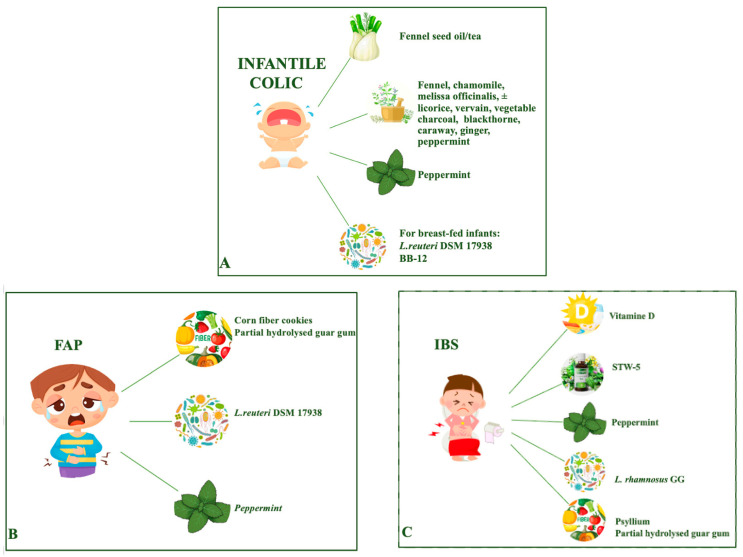
Evidence of nutraceuticals’ efficacy in infantile colic (**A**), functional abdominal pain (**B**), irritable bowel syndrome (**C**).

Nutraceuticals are often considered by parents to treat their child with infantile colic and abdominal pain disorders. However, there is a very limited number of studies assessing the efficacy and tolerance of herbs, spices, and nutritional supplements in children with these conditions. Growing evidence of efficacy in reducing infant colic episodes and crying time is emerging for fennel (seeds oil or tea or in different multiple herbal extracts), *Lactobacillus reuteri* DSM 17938, and *Bifidobacterium lactis* BB-12 in breast-fed infants. In children and adolescents with functional abdominal pain or irritable bowel syndrome, a reduction in pain was reported in patients supplemented with *Lactobacillus reuteri* DSM 17938 or *Lactobacillus rhamnosus* GG and in two studies using peppermint oil capsules or psyllium fibers; the benefits of corn fiber cookies, partial hydrolyzed guar gum, a specific multiple herbal extract (STW-5), or vitamin D supplementation were demonstrated only in one study for each product (Figure 1). No recommendation can currently be provided for other nutraceuticals and possible adverse effects should always be considered. Moreover, current pediatric studies present small sample sizes; heterogeneity in the population recruited, the product and dosage used, and outcome measures; and lack long-term assessment of efficacy and safety.
